# Lin4Neuro: a customized Linux distribution ready for neuroimaging analysis

**DOI:** 10.1186/1471-2342-11-3

**Published:** 2011-01-25

**Authors:** Kiyotaka Nemoto, Ippeita Dan, Christopher Rorden, Takashi Ohnishi, Daisuke Tsuzuki, Masako Okamoto, Fumio Yamashita, Takashi Asada

**Affiliations:** 1 Department of Psychiatry, Graduate School of Comprehensive Human Sciences, University of Tsukuba, 1-1-1 Ten-nodai Tsukuba, Ibaraki, 305-8575, Japan; 2 Functional Brain Science Lab., Center for Development of Advanced Medical Technology, Jichi Medical University, 3311-1 Yakushiji Shimotsuke, Tochigi, 329-0498, Japan; 3 GeorgiaState/GeorgiaTech Center for Advanced Brain Imaging, Georgia Institute of Technology, 831 Marietta Street, Atlanta, GA 30332, USA; 4 Department of Psychosomatic Research, National Institute of Mental Health, National Center of Neurology and Psychiatry, 4-1-1 4-1-1 Ogawa-Higashi Kodaira, Tokyo, 187-8553, Japan; 5 Graduate School of System and Information Engineering, University of Tsukuba, 1-1-1 Ten-nodai Tsukuba, Ibaraki, 305-8575, Japan; 6 Animal Global Health Program, Obihiro University of Agriculture and Verterinary Medicine, Inada-cho, Obihiro, Hokkaido, 080-8555, Japan

## Abstract

**Background:**

A variety of neuroimaging software packages have been released from various laboratories worldwide, and many researchers use these packages in combination. Though most of these software packages are freely available, some people find them difficult to install and configure because they are mostly based on UNIX-like operating systems. We developed a live USB-bootable Linux package named "Lin4Neuro." This system includes popular neuroimaging analysis tools. The user interface is customized so that even Windows users can use it intuitively.

**Results:**

The boot time of this system was only around 40 seconds. We performed a benchmark test of inhomogeneity correction on 10 subjects of three-dimensional T1-weighted MRI scans. The processing speed of USB-booted Lin4Neuro was as fast as that of the package installed on the hard disk drive. We also installed Lin4Neuro on a virtualization software package that emulates the Linux environment on a Windows-based operation system. Although the processing speed was slower than that under other conditions, it remained comparable.

**Conclusions:**

With Lin4Neuro in one's hand, one can access neuroimaging software packages easily, and immediately focus on analyzing data. Lin4Neuro can be a good primer for beginners of neuroimaging analysis or students who are interested in neuroimaging analysis. It also provides a practical means of sharing analysis environments across sites.

## Background

The flourishing development of the neuroimaging research field has been in part supported by a wealth of open-source software packages released from various laboratories worldwide. Besides the obvious merit of being available for free, they are often equipped with cutting-edge analytical tools that are not included in commercial software packages, allowing researchers to perform state-of-the-art neuroimaging analyses.

These packages are usually developed for a specific purpose, but many researchers use them in combination. For example, Acosta-Cabronero et al. [1] demonstrated that the combination of skull stripping with BET2 [2] and intensity inhomogeneity correction with N3 [3] improved the accuracy of the gray matter segmentation of SPM5 [4].

Conventionally, UNIX or UNIX-like operating systems such as Linux have served as platforms for developing most of these software packages. However, not all researchers in the field of neuroimaging are familiar with those versatile yet recondite operating systems. This is especially so given the interdisciplinary development of neuroimaging research, which inevitably entails the influx of researchers without Linux experience. Though installing Linux has been getting much easier, many still find it difficult to set up and maintain an effective Linux environment for neuroimaging software packages. For example, if a Windows user tries to use a neuroimaging software package that works on Linux, he/she has to start by installing Linux, then install the neuroimaging analysis software packages, and still has to struggle with the configuration processes. As a result, many researchers spend vast amounts of time merely setting up a neuroimaging analysis environment before they can actually start analyzing.

LONI pipeline offers a solution to these problems, enabling one to access various software packages released from different laboratories in different environments from a unified user interface [5,6]. However, LONI pipeline is a server-client solution, and, if one wants to use it on a stand-alone computer, one must install all of the software packages beforehand. Recently, a project named Nipype [7] has provided a uniform interface for existing neuroimaging software packages, and facilitates interaction between these packages within a single workflow. This is an excellent tool, which encourages interactive exploration of algorithms from different packages, but users still have to install different packages beforehand. In this respect, there have not been any simple solutions by which users can use Linux-based software packages immediately.

One alternative is to use "live Linux," which contains a bootable computer operating system. Live Linux is unique in that it has the ability to run a complete, modern operating system on a computer without using installed storage such as a hard disk drive. It is very useful for several reasons. First, many software packages can be installed beforehand in a CD, DVD, or USB flash drive so that one can use them immediately upon booting a computer from live Linux. Second, it does not have to be installed on the computer's hard disk, making it portable so that one can recreate one's environment on any computer. Third, live Linux can also be installed on a hard disk with only a few mouse clicks: this is the most time-effective way to set up a neuroimaging analysis system.

If we provide a live Linux system in which neuroimaging software packages have already been installed, it will not only save time for researchers, but also expand the horizons of the neuroimaging field by allowing the easy setup of neuroimaging processing environments. Therefore, we developed a live Linux package named "Lin4Neuro."

## Implementation

### Specifications of Lin4Neuro

We employed Ubuntu Linux 10.04 Desktop LTS (Long Term Support) 32/64bit version [8] as the base operating system. We chose Ubuntu mainly for the following four reasons: First, several neuroimaging analysis software packages have guidelines for installation on Ubuntu; additionally, a useful repository named NeuroDebian [9] is provided so that one can easily install and update neuroscience-related packages on Ubuntu. Second, a utility named Remastersys, available on Ubuntu, enables one to generate a live Linux environment. Third, Ubuntu is highly flexible, and can be optimized for a particular type of analysis. For example, many neuroimaging analysis software packages require a lot of memory. Ubuntu's versatile memory management tools can minimize the memory consumption of the operating system, and allow one to make the best use of memory for neuroimaging software packages. Finally, the Ubuntu installer is sophisticated, allowing the easy installation of Ubuntu on a hard disk.

As for neuroimaging analysis software to be incorporated into Lin4Neuro, we chose the following:

1. 3D Slicer developed by the Brigham and Women's Hospital, Inc. U.S.A. [10]

2. AFNI developed by the Scientific and Statistical Computing Core of the NIMH Intramural Research Program, U.S.A. [11,12]

3. AMIDE developed by Andy Loening, U.S.A. [13,14]

4. Caret developed by The Van Essen Laboratory at the Washington University School of Medicine in Saint Louis, Missouri, USA. [15,16]

5. FSL developed by FMRIB, Oxford, United Kingdom [17,18]

6. ITK developed by the Insight Software Consortium, U.S.A. [19]

7. LIPSIA developed by the Max-Planck-Institute for Human Cognitive and Brain Sciences in Leipzig, Germany. [20,21]

8. MINC software packages including MNI-N3 developed by the McConnell Brain Imaging Center of the Montreal Neurological Institute, McGill University, Canada [22]

9. MRIConvert developed by Jolinda Smith, the Lewis Center for Neuroimaging at the University of Oregon, U.S.A. [23]

10. MRIcron developed by Chris Roden, U.S.A [24,25]

11. Virtual MRI by Thomas Hacklaender, Germany [26,27]

12. (x)Medcon developed by Eric Nolf, Belgium. [28]

Table 1 describes the function of the each package listed above. They are distributed under the GNU GPL (General Public License), BSD (Berkeley Software Distribution) license, and under any license which states that the packaged software can be redistributed for research purposes provided there is no financial return. We did not include SPM (Statistical Parametric Mapping) developed by Wellcome Trust Centre for Neuroimaging because SPM requires Matlab, which is a commercial software package.

**Table 1:** Neuroimaging software packages included in Lin4Neuro

Package Name	Description
3D Slicer	Tools for the segmentation, registration, and three-dimensional visualization of multi-modal image data. The package includes image analysis algorithms for diffusion tensor imaging, functional magnetic resonance imaging, and image-guided therapy.
AFNI	An environment for processing and displaying functional MRI data. It provides a complete analysis tool chain, including 3D cortical surface models and mapping of volumetric data (SUMA).
Amide	A tool for viewing and analyzing medical image data sets. It includes the simultaneous handling of multiple data sets imported from a variety of file formats, image fusion, 3D region of interest drawing and analysis, volume rendering, and rigid body alignments.
Caret	A tool which enables the user to create, view, and manipulate surface reconstructions of the cerebral and cerebellar cortex. It also displays experimental data on surfaces and volumes.
FSL	A comprehensive library of image analysis and statistical tools for fMRI, MRI, and DTI brain imaging data. Implementation of standard GLM analysis, white matter tractography, tissue segmentation, affine and non-linear co-registration, and independent component analysis.
ITK	Providing segmentation and registration algorithms in two, three, and more dimensions.
LIPSIA	A tool for data processing and evaluation of functional magnetic resonance images.
MINC-tools	Tools to manipulate MINC files.
MNI-N3	A tool for unsupervised correction of radio frequency (RF) field inhomogeneities in MR volumes.
MRIConvert	A medical image file conversion utility that converts DICOM files to NIfTI 1.1, Analyze 7.5, SPM99/Analyze, BrainVoyager, and MetaImage volume formats.
MRIcron	A GUI-based visualization and analysis tool for (functional) MRI. It can also draw anatomical regions-of-interest (ROI), or lesion mapping, as well as basic analysis of functional time series. In addition to 'mricron', the package also provides 'dcm2nii', which supports converting DICOM and PAR/REC images into the NIfTI format and 'npm' for non-parametric data analysis.
Virtual MRI	A realistic simulation of an MRI scanner. For the user it should be possible to change all the relevant settings of the virtual scanner and to adapt them to the expected pathology.
(x)Medcon	A tool to convert medical images. Supported formats are: Acr/Nema 2.0, Analyze (SPM), Concorde/μPET, DICOM 3.0, CTI ECAT 6/7, NIfTI-1, InterFile3.3, and PNG or Gif87a/89a.

First we installed Ubuntu and the software packages listed above, and set up the configuration procedures necessary to run them stably. We also implemented a tutorial and sample dataset showing how to use the packages included in Lin4Neuro.

**Figure 1:**
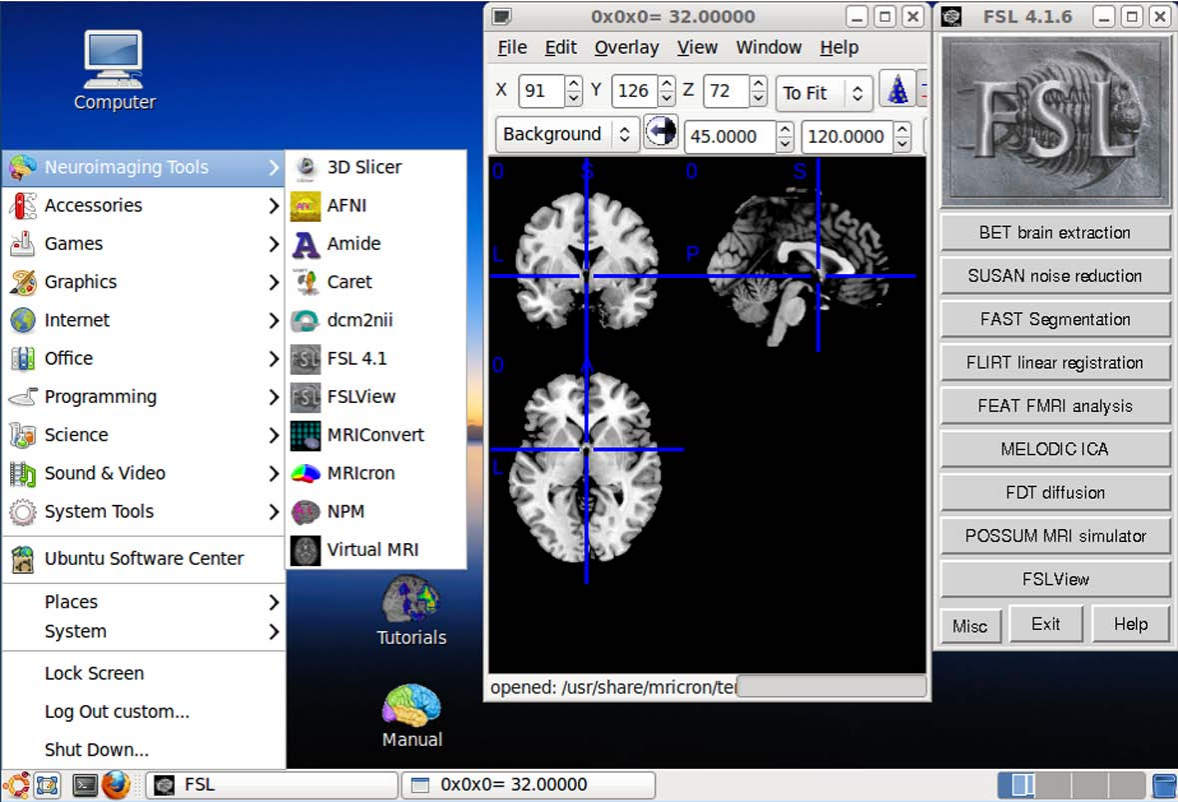
****Screenshot of Lin4Neuro****. User interface of Lin4Neuro was customized so that even Windows users can use this system intuitively. Shortcuts for tutorials and the manual are provided on the desktop, so users can reach the necessary resources easily with a few clicks.

Then, we customized the desktop interface to have a Windows-like appearance so that typical Windows users could easily familiarize themselves with the Linux environment (Figure 1). Following the customization, we made a distributable copy using Remastersys [29]. Finally, live USB flash drives were made using the disk creator utility in Ubuntu.

Lin4Neuro will be distributed as an iso image; Windows users can create live USB flash drives with a software package called "Universal USB Installer" [30]. It takes only 40 seconds to boot on a mid-range personal computer with a Core2Quad Q6600 CPU running at 2.33 GHz with 4GB memory. Upon booting from the live USB flash drive, the software packages listed above are ready to use on most personal computers. In addition, one can install Lin4Neuro on a hard disk from the live USB flash drive. After being installed, Lin4Neuro can be customized, and even personalized, using the Remastersys program.

### Application of Lin4Neuro

The easy installation and system portability featured in Lin4Neuro not only allow one to access various neuroimaging packages, but also make the neuroimaging-processing environment sharable. One example of where such merits would be extremely beneficial is in multicenter studies on structural MR images, in which imaging data among several different research groups must be integrated through a common protocol. Obviously, Lin4Neuro is beneficial in realizing a common platform that can be shared among different research groups. Moreover, it provides a series of preprocessing tools that are often required for cross-center data comparison. Since it is not feasible that all the groups possess the same imaging facility, neuroimaging data should be more or less corrected for inhomogeneity to the degree that they are comparable. Intensity inhomogeneity of MR images is known to affect the precision of the results of segmentation [1,31,32]. Thus, it is important that the same protocols for imaging data preprocessing are shared within and across each institute, and Lin4Neuro provides an ideal environment for this purpose. In the following example, we will demonstrate how the inhomogeneity correction is implemented in this system.

**Figure 2:**
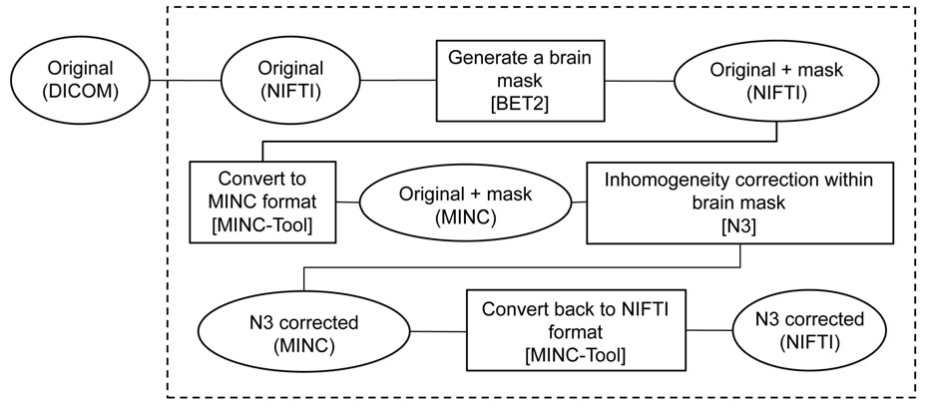
**Schematic of inhomogeneity correction procedure performed on Lin4Neuro**. Input images in the DICOM format were sequentially processed by MRIConvert, BET2, MINC-Tool, N3, and MINC-Tool to generate inhomogeneity-corrected images in NIFTI-1 format. Procedures within the dashed box were automated with a short shell script in Lin4Neuro. Open circles indicate formats. Open squares indicate software used for the procedures.

According to the method by Acosta-Cabronero et al., [1] we prepared a batch script, which processes the following in series: First, we stripped the skull and made a mask of the brain using BET2, included in FSL. Second, the processed images were converted to MINC format with the MINC-Tool included in the MINC software package. Third, we performed inhomogeneity correction using N3, included in the MINC software packages. The brain mask generated by BET2 was used to identify the region to which N3 was applied. Finally, the inhomogeneity-corrected images were converted back to NIFTI-1 format with the MINC-Tool, and made ready for segmentation with software packages such as SPM or FSL. A schematic of the processing is shown in Figure 2.

## Results

Using the batch script above, we performed a benchmark test of Lin4Neuro under several system conditions. We performed the batch analyses described above on 10 subjects of three-dimensional T1-weighted MRI scans from the freely available IXI dataset [33].

The results are summarized in Table 2. First of all, the processing time of the USB-boot Lin4Neuro was as fast as that of the HDD-boot. This proved that USB-booting of Lin4Neuro does not compromise on speed. The processing time of the 64bit Lin4Neuro was around 1.5 minutes (15%) faster than the 32bit time with the same hardware, proving that the system and software packages were well optimized for the 64bit architecture. We also installed Lin4Neuro on a VMware player [34], a virtualization software package that emulates the Linux environment on a Windows-based operation system. Although the processing speed was slower than that under other conditions, it remained comparable. It should be noted that memory and cores of CPU were limited for the virtual machine.

**Table 2:** Benchmark results of the inhomogeneity correction script

Booted from	32/64bit	CPU	CPU frequency	Number of cores	RAM	Processing time of the intensity inhomogeneity correction script
*Difference between USB-boot and HDD-boot*						
HDD	64bit	Core2Quad Q6600	2.4GHz	4	4GB	11min 1.38sec
USB flash drive	64bit	Core2Quad Q6600	2.4GHz	4	4GB	11min 1.30sec
*Difference between 32bit and 64bit*						
USB flash drive	32bit	corei7 L640	2.13GHz	4	3GB	11min 13.01sec
USB flash drive	64bit	corei7 L640	2.13GHz	4	4GB	9min 47.68sec
Difference between 32bit and 64bit on Virtualization software*						
VMware player	32bit	corei7 L640	2.13GHz	2	1GB	11min 36.64sec
VMware player	64bit	corei7 L640	2.13GHz	2	1GB	12min 1.23sec

The processing time of intensity inhomogeneity correction for 10 three-dimensional T1-weighted MRI scans was measured using the "time" command in Linux.

*VMware player was executed on 64 bit Windows 7 platform with core i7 L640 (4 cores) with 4GB RAM, but the number of cores and RAM size were reduced to 2 and 1GB, respectively, due to constraints of the emulation mode.

## Discussion

Those who are involved in neuroimaging analysis have surely experienced spending an inordinate amount of time setting up a system environment before analyzing data. Still, they are lucky in the sense that they have managed to set it up: how many researchers struggle in vain, giving up before they are able to analyze their data? Setting up a system environment should not be a rite of passage into "neuroimaginghood." With Lin4Neuro in one's hand, one can access neuroimaging software packages easily and immediately focus on analyzing data. This approach will also benefit developers of software packages since the pre-installation of software will give many potential users opportunities to try them.

As an application of Lin4Neuro, we compared the processing time of intensity inhomogeneity correction under different system conditions. Surprisingly, the results showed that the USB-boot live Linux system is as capable as the hard-disk-installed Linux system. This means that one is able to achieve a versatile Linux environment for neuroimaging analyses by simply booting Lin4Neuro from a USB flash drive, omitting complicated procedures for installing and configuring Linux and Linux-based neuroimaging software packages. In addition, the current study also demonstrated the possibility of utilizing virtualization. Although full use of hardware power cannot be achieved, virtualization may provide a practical solution for realizing a neuroimaging Linux environment on a Windows PC. Thus, using live Linux or virtualization software, one does not have to prepare new hardware but can make the best use of one's current hardware.

It is noteworthy that many neuroimaging software packages have command-line utilities. This makes the combining of functions of different software packages easy, and the processing of images automatic. Providing a common analytical platform with Lin4Neuro facilitates such integrative protocols across packages. In the future, we plan to incorporate Nipype into this system, which will provide an environment that encourages interactive exploration of algorithms from different packages, eases the design of workflows within and between packages, and reduces the learning curve necessary to use different packages.

Recently, multi-center studies have become increasing common. Accordingly, quality control of imaging data is gaining much more importance. For example, Alzheimer's disease Neuroimaging Initiative (ADNI) standardized the MRI protocol, post-acquisition corrections, and phantom-based monitoring of all scanners across sites and platforms [35]. Lin4Neuro provides a practical means of sharing the analysis environment across sites. Once this system is installed on a hard disk, one can customize the environment and re-create distributable copies. By doing so, each center can share a neuroimaging analysis environment for specific research. Moreover, one can replicate the image processing protocols easily by sharing the scripts which describe how images are processed. From a different perspective, it should also be noted that Lin4Neuro on a USB flash drive can serve as an installer for a Linux operating system and Linux-based neuroimaging software packages. With a slight modification of the BIOS (Basic Input/Output System) settings to enable booting from the USB flash disk, one can easily realize an optimized Linux environment for neuroimaging analyses on a new personal computer.

With the interdisciplinary development of neuroimaging research, not only neuroscientists but also many researchers from different fields are participating in neuroimaging research. Thus, it is becoming more important to establish a platform in which one can easily access various neuroimaging analysis software packages. Lin4Neuro could be a good primer for beginners of neuroimaging analysis or students who are interested in neuroimaging analysis.

In the current release, we limited the software packages incorporated into the system to only those that are frequently used. However, there are many software packages which are redistributable on the internet. Recently, a website named NITRC (The Neuroimaging Informatics Tools and Resources Clearinghouse) has been established and is open to public, enabling us to find many useful neuroimaging software packages [36,37]. In the future, it might be possible to include a wide variety of redistributable software packages based on information from NTIRC. Though we cannot include commercial software packages or software packages that require license keys, installing Lin4Neuro on a hard disk will enable the user to install any Linux-based software packages. Specifically, SPM, one of the most influential neuroimaging software packages, which is a freeware package itself but dependent on Matlab, is currently excluded from Lin4Neuro. However, since a standalone SPM which does not a require MATLAB license is under development [38], hopefully it may be included in Lin4Neuro in a future release.

As our system includes many well-known software packages, some may argue that the contributions of this study are not original. However, the genuine originality of our study lies in the creation of a unique and integrative neuroimaging environment. In this respect, Lin4Neuro plays a role as a portal for neuroimaging software packages and keeps researchers from getting lost in vast amounts of information. Lin4Neuro is available on the Internet [39]. Since this system has an interactive nature, we hope that, through interaction among users, Lin4Neuro will become more sophisticated and easier to use, and consequently contribute to the further development of the neuroimaging field.

## Conclusion

With Lin4Neuro in one's hand, one can easily establish a neuroimaging analysis environment. Lin4Neuro is beneficial for beginners of neuroimaging analysis or students who are interested in neuroimaging analysis. It can also benefit experts in that it provides a practical means of sharing analysis environments across computers or even sites.

## Availability and requirements

Project name: Lin4Neuro

Project home page: http://www.nemotos.net/lin4neuro/

Operating system: Linux (based on Ubuntu 10.04 LTS)

Program language: N/A

Other requirements: VMware or VirtualBox (Windows)

License: GNU General Public License (GPL) Restrictions to use by non-academic: Lin4Neuro is intended for research purpose only.

## Competing interests

The authors declare that they have no competing interests.

## Authors' contributions

KN designed the system, carried out the benchmark test, and wrote the manuscript. ID played a subsidiary role in the study design and writing the manuscript. CR prepared the tutorial and sample dataset implemented in Lin4Neuro. DT and MO tested the system and helped to draft the manuscript. TO, FY, and TA participated in the study design and tested the system. All authors read and approved the final manuscript.

## Pre-publication history

The pre-publication history for this paper can be accessed here:

http://www.biomedcentral.com/1471-2342/11/3/prepub
